# Supplementary material for: A Critical View of the Application of the APEX Software (Aqueous Photochemistry of Environmentally-Occurring Xenobiotics) to Predict Photoreaction Kinetics in Surface Freshwaters
Source: Molecules. 2019 Dec 18;25(1):9. doi: 10.3390/molecules25010009 (PMC7017383; doi:10.3390/molecules25010009)
Supplement: Supplementary file 1 [file molecules-25-00009-s001.zip › 3.2.4_gcc-4.4.0.zip!/3.2.4_gcc-4.4.0/3.2.4_gcc-4.4.0/doc/octave/html/interpreter/Script-Files.html]

Script Files - Untitled


Next: Function Handles Inline Functions and Anonymous Functions,
Previous: Function Files,
Up: Functions and Scripts


---

### 11.8 Script Files

A script file is a file containing (almost) any sequence of Octave
commands. It is read and evaluated just as if you had typed each
command at the Octave prompt, and provides a convenient way to perform a
sequence of commands that do not logically belong inside a function.

Unlike a function file, a script file must *not* begin with the
keyword `function`. If it does, Octave will assume that it is a
function file, and that it defines a single function that should be
evaluated as soon as it is defined.

A script file also differs from a function file in that the variables
named in a script file are not local variables, but are in the same
scope as the other variables that are visible on the command line.

Even though a script file may not begin with the `function`
keyword, it is possible to define more than one function in a single
script file and load (but not execute) all of them at once. To do
this, the first token in the file (ignoring comments and other white
space) must be something other than `function`. If you have no
other statements to evaluate, you can use a statement that has no
effect, like this:

```
     # Prevent Octave from thinking that this
     # is a function file:
     
     1;
     
     # Define function one:
     
     function one ()
       ...
```

To have Octave read and compile these functions into an internal form,
you need to make sure that the file is in Octave's load path
(accessible through the `path` function), then simply type the
base name of the file that contains the commands. (Octave uses the
same rules to search for script files as it does to search for
function files.)

If the first token in a file (ignoring comments) is `function`,
Octave will compile the function and try to execute it, printing a
message warning about any non-whitespace characters that appear after
the function definition.

Note that Octave does not try to look up the definition of any identifier
until it needs to evaluate it. This means that Octave will compile the
following statements if they appear in a script file, or are typed at
the command line,

```
     # not a function file:
     1;
     function foo ()
       do_something ();
     endfunction
     function do_something ()
       do_something_else ();
     endfunction
```

even though the function `do_something` is not defined before it is
referenced in the function `foo`. This is not an error because
Octave does not need to resolve all symbols that are referenced by a
function until the function is actually evaluated.

Since Octave doesn't look for definitions until they are needed, the
following code will always print bar = 3 whether it is typed
directly on the command line, read from a script file, or is part of a
function body, even if there is a function or script file called
bar.m in Octave's path.

```
     eval ("bar = 3");
     bar
```

Code like this appearing within a function body could fool Octave if
definitions were resolved as the function was being compiled. It would
be virtually impossible to make Octave clever enough to evaluate this
code in a consistent fashion. The parser would have to be able to
perform the call to `eval` at compile time, and that would be
impossible unless all the references in the string to be evaluated could
also be resolved, and requiring that would be too restrictive (the
string might come from user input, or depend on things that are not
known until the function is evaluated).

Although Octave normally executes commands from script files that have
the name file.m, you can use the function `source` to
execute commands from any file.

— Built-in Function: **source** (file)  
> Parse and execute the contents of file. This is equivalent to
> executing commands from a script file, but without requiring the file to
> be named file.m.
